# Sporogony of four *Haemoproteus* species (Haemosporida: Haemoproteidae), with report of *in vitro* ookinetes of *Haemoproteus hirundinis*: phylogenetic inference indicates patterns of haemosporidian parasite ookinete development

**DOI:** 10.1186/s13071-019-3679-1

**Published:** 2019-08-28

**Authors:** Carolina Romeiro Fernandes Chagas, Dovilė Bukauskaitė, Mikas Ilgūnas, Rasa Bernotienė, Tatjana Iezhova, Gediminas Valkiūnas

**Affiliations:** Institute of Ecology, Nature Research Centre, Akademijos 2, LT-08412 Vilnius, Lithuania

**Keywords:** *Haemoproteus*, Birds, Cytochrome *b* gene, Sporogony, Vectors, *Culicoides nubeculosus*

## Abstract

**Background:**

*Haemoproteus* (*Parahaemoproteus*) species (Haemoproteidae) are widespread blood parasites that can cause disease in birds, but information about their vector species, sporogonic development and transmission remain fragmentary. This study aimed to investigate the complete sporogonic development of four *Haemoproteus* species in *Culicoides nubeculosus* and to test if phylogenies based on the cytochrome *b* gene (*cytb*) reflect patterns of ookinete development in haemosporidian parasites. Additionally, one *cytb* lineage of *Haemoproteus* was identified to the species level and the *in vitro* gametogenesis and ookinete development of *Haemoproteus hirundinis* was characterised.

**Methods:**

Laboratory**-**reared *C. nubeculosus* were exposed by allowing them to take blood meals on naturally infected birds harbouring single infections of *Haemoproteus belopolskyi* (*cytb* lineage hHIICT1), *Haemoproteus hirundinis* (hDELURB2), *Haemoproteus nucleocondensus* (hGRW01) and *Haemoproteus lanii* (hRB1). Infected insects were dissected at intervals in order to detect sporogonic stages. *In vitro* exflagellation, gametogenesis and ookinete development of *H. hirundinis* were also investigated. Microscopic examination and PCR-based methods were used to confirm species identity. Bayesian phylogenetic inference was applied to study the relationships among *Haemoproteus* lineages.

**Results:**

All studied parasites completed sporogony in *C. nubeculosus*. Ookinetes and sporozoites were found and described. Development of *H. hirundinis* ookinetes was similar both *in vivo* and *in vitro*. Developing ookinetes of this parasite possess long outgrowths, which extend longitudinally and produce the apical end of the ookinetes. A large group of closely related *Haemoproteus* species with a similar mode of ookinete development was determined. Bayesian analysis indicates that this character has phylogenetic value. The species identity of *cytb* lineage hDELURB2 was determined: it belongs to *H. hirundinis*.

**Conclusions:**

*Culicoides nubeculosus* is susceptible to and is a likely natural vector of numerous species of *Haemoproteus* parasites, thus worth attention in haemoproteosis epidemiology research. Data about *in vitro* development of haemoproteids provide valuable information about the rate of ookinete maturation and are recommended to use as helpful step during vector studies of haemosporidian parasites, particularly because they guide proper dissection interval of infected insects for ookinete detection during *in vivo* experiments. Additionally, *in vitro* studies readily identified patterns of morphological ookinete transformations, the characters of which are of phylogenetic value in haemosporidian parasites.

## Background

Avian haemosporidian parasites (*Plasmodium*, *Haemoproteus, Leucocytozoon* and *Fallisia*) are widespread and remarkably diverse; this diversity frequently exceeds that of avian hosts [1–5]. During the past ten years, it has been shown that several *Haemoproteus* species can be harmful to their avian hosts, compromising their health and even causing mortality, especially in non-adapted birds [6–9]. This calls for additional studies aimed at a better understanding of pathogen transmission. Blood-sucking insects can also be negatively affected and even killed by *Haemoproteus* parasites after feeding on heavily infected blood (when > 1% of gametocytes are mature) [10–12].

Many studies have addressed parasite diversity and the phylogenetic relationships of haemosporidian parasites of various avian hosts [4, 5, 13–18], but information about vector competence and patterns of sporogonic development of these pathogens remains insufficient. *Haemoproteus* (*Parahaemoproteus*) species (Haemosporida: Haemoproteidae) are cosmopolitan in countries with warm and temperate climates [2, 3]. These blood parasites are transmitted by biting midges of the Ceratopogonidae [2]. Recently, several studies addressed *Haemoproteus* spp. sporogonic development in several species of *Culicoides* biting midges [19–23]. However, due to the big variety of *Haemoproteus* species and the scarcity of detailed information about patterns of sporogonic development in the majority of the described species, understanding haemoproteid parasite sporogony remains essential for better understanding the biology of haemosporidians. This calls for additional experimental and field vector research.

The presence of haemosporidian parasites in wild-caught insects has been frequently reported in many parts of the world using PCR-based diagnostics [24–30]. However, this methodology alone does not provide information about patterns of sporogonic development. Additionally, identification of PCR-positive insects does not necessarily show that sporogony is completed and sporozoites (infective stage for birds) reach salivary glands. On the other hand, the observation of infective sporozoite stages in salivary glands (accessed through insect dissection and microscopical analysis) provides stronger evidence about the possible vectorial capability of blood-sucking insects. Experimental infections not only allow one to follow parasite development and morphologically characterize each sporogonic stage, but also to confirm the presence of sporozoites in the salivary glands [22, 23, 31]. Laboratory-reared *Culicoides nubeculosus* can be used for conducting such experimental infections with *Haemoproteus* parasites. The methodology for rearing this biting midge in the laboratory was developed over 40 years ago [32], and so far, this insect has been shown to be susceptible to eight *Haemoproteus* species [22, 23].

Phylogenetic and genomics research are in progress in wildlife haemosporidians studies [33, 34]. However, it remains unclear if and how numerous available phylogenies, which are based on DNA sequences, reflect patterns in biology of haemosporidians. In other words, it remains insufficiently understood what the well-supported phylogenetic clades indicate in regard to the biology of the parasites. Phylogenies based on mitochondrial genes indicate haemosporidian parasite-vector relationships [22, 35] and it is probable that they also can be used for more delicate understanding of sporogony in haemosporidians. However, this issue has not been addressed in haemosporidian research.

Interestingly, the exflagellation, gametogenesis and development of ookinetes of *Haemoproteus* species can be readily induced *in vitro* when mature gametocytes are exposed to air. This process is easy to initiate with *Haemoproteus* parasites because they exflagellate *in vitro* without need of any vector-related gut factors, which is not the case in *Plasmodium* parasites of mammals [36] and birds [2]. This provides opportunities to access initial sporogonic stages (gametes, zygotes, ookinetes) in standard *in vitro* conditions for comparative studies. *In vitro* exflagellation experiments were successfully used to characterize gametogenesis and ookinete development of several *Haemoproteus* species [2, 37, 38]; they were used in haemosporidian parasite hybridization studies [39–41] and genomic research when large amount of pure parasite DNA is needed [42]. This methodology can also be applied in haemosporidian vector studies, since it provides information about ookinete morphological transformation and development rate. The latter character provides important guidance in determining the optimal dissection time of infected insects for the detection of mature ookinetes *in vivo* [2, 31].

Parasite distribution is influenced by numerous factors [2, 43]. In haemosporidians, not only parasite, vector and bird community factors are important in epidemiology [44], but the effect of environmental and other ecological variables are fundamental in parasite prevalence and distribution [45–47]. Identifying the factors, which limit parasite transmission, is important for better understanding disease epidemiology and development of preventive measures. This issue is particularly sensitive to address regarding several widespread *Haemoproteus* parasites, which are present and prevalent in European birds, but are not transmitted in Europe, for example *Haemoproteus hirundinis*, *Haemoproteus payevskyi* and *Haemoproteus nucleocondensus* [2, 48].

This study investigated the sporogonic development of four *Haemoproteus* (*Parahaemoproteus*) parasites: *Haemoproteus belopolskyi* (cytochrome *b* gene lineage hHIICT1), *H. hirundinis* (hDELURB2), *H. nucleocondensus* (hGRW01) and *Haemoproteus lanii* (hRB1). The main aim was to follow complete sporogony of these parasites in the biting midge *Culicoides nubeculosus*, which is widespread in Europe [49]. Additionally, we investigated the *in vitro* exflagellation, gametogenesis and ookinete development of *H. hirundinis* (hDELURB2) and compared this process with the same features reported in other avian haemoproteids. The phylogenetic relationships were inferred among *Haemoproteus* species, for which vectors and ookinete development *in vitro* have been identified. Available information about transmission of these parasites in Europe is discussed.

## Methods

### Blood sampling and microscopic analysis

Experiments were carried out at the Ventės Ragas Ornithological Station, Lithuania (55°20′28.1″N, 21°11′25.3″E) in May and June 2018. Birds were captured using mist nets, ‘Zigzag’ traps and a funnel trap. Blood samples were collected from the brachial vein (~30 µl) using heparinized microcapillaries. A small drop of blood was used to prepare blood smears which were immediately air-dried using a battery-powered fan, fixed in absolute methanol and stained with Giemsa [2]. Remaining blood was stored in SET buffer (0.05 M Tris, 0.15 M NaCl, 0.5 M EDTA, pH 8.0) for DNA extraction. Blood smears were examined using an Olympus BX-43 light microscope equipped with Olympus SZX2-FOF digital camera and QCapture Pro 6.0, Image Pro Plus (Olympus, Tokyo, Japan) imaging software. The analysis consisted of screening each slide for 15–20 min at low magnification (×400) and at least 100 fields at high magnification (×1000). Parasitaemia was determined by actual counting of the number of mature gametocytes per 1000 red blood cells or per 10,000 red blood cells when low parasitaemia was present (≤ 0.1%) [50]. Parasites were morphologically identified according to Valkiūnas [2] and available literature.

### *In vitro* exflagellation and ookinete development rate

One individual northern house martin *Delichon urbicum*, with parasitaemia of mature gametocytes of approximately 0.2% was selected as a *H. hirundinis* (hDELURB2) gametocyte donor to observe *in vitro* exflagellation, gametogenesis and ookinete development. The experiment followed the methodology described by Valkiūnas [2] and Valkiūnas et al. [40]. Briefly, approximately 100 µl of blood was collected from the brachial vein and immediately mixed with a 3.7% solution of sodium citrate in a microtube Eppendorf type, in the proportion of 4 parts blood to 1 part sodium citrate solution. This mixture was maintained opened in a humid chamber at room temperature (~21 °C). Blood smears were prepared at intervals of 1, 3, 5, 10, 15, 30 and 45 min and at 1, 2, 3, 4, 6, 8, 10, 12 and 24 h after exposure of blood to air (EBA). The blood mixture was gently homogenised before blood smear preparation. Blood smears were air dried, fixed, stained and analysed as described for blood films above. The donor bird was released after blood collection. Representative preparations for examination of exflagellation, gametogenesis and ookinete development were deposited in the Nature Research Centre, Vilnius, Lithuania, under the accession numbers 49134–49147 NS.

### Experimental design of sporogonic development *in vivo*

After microscopic examination of the blood smears, birds harbouring single *Haemoproteus* infections with low parasitaemia (≤ 0.1% mature gametocytes) were selected as donors (see Bukauskaitė et al. [20] for details of exposure). One individual of the following bird species was exposed to biting midges: the icterine warbler *Hippolais icterina*, the northern house martin *Delichon urbicum*, the great reed warbler *Acrocephalus arundinaceus* and the red-backed shrike *Lanius collurio* were infected with *Haemoproteus belopolskyi* (hHIICT1), *Haemoproteus hirundinis* (hDELURB2), *Haemoproteus nucleocondensus* (hGRW01) and *Haemoproteus lanii* (hRBS4), respectively. All birds were immediately released after the experiment.

Laboratory-reared *Culicoides nubeculosus* biting midges were kept in small cardboard boxes covered with fine silk mesh. Each box hosted approximately 80 biting midge individuals. Boxes with insects were gently pressed against the pectoral muscle of birds on a feather-free area. Insects were allowed to take a blood meal throughout the silk mesh for about 40 min. Biting midges where then transferred to a bigger cage (12 × 12 × 12 cm^3^) made of wire and fine silk mesh and males along with non-fed females were removed. Engorged females were kept at 24.8 ± 0.5 °C, 60 ± 4% humidity and controlled light-dark photoperiod (17:7 h). Insects were fed daily using cottons pads moistened with 10% sugar solution placed on the top of each cage.

### Insect dissection and parasite preparation

Experimentally-infected biting midges were dissected at set of intervals to prepare ookinete preparations (which were made between 4 and 12 h post-exposure), oocyst preparations (between 2 and 7 days post-exposure) and sporozoite preparations (between 6 and 9 days post-exposure). Dissection needles were disinfected after each dissection using fire to prevent contamination. Before dissection, insects were anesthetized using 96% ethanol vapour from a moistened cotton-wool ball.

For ookinete preparations, midguts were extracted and gently crushed on glass slides; thin smears were prepared, fixed and stained as described above for blood slides. To visualize oocysts, temporary preparations were made by isolation of midgut, which was placed on a glass slide and covered with a cover-slip. A drop of 2% mercurochrome solution was used to stain midguts and differentiate oocysts. To prepare sporozoite preparations, biting midge salivary glands were extracted and gently crushed to prepare small thin smears that were fixed in absolute methanol and stained with Giemsa using a 4% solution for 1 h. Residual parts of all dissected insects were fixed in 96% ethanol and used for PCR-based analysis to confirm the presence of corresponding parasite lineage.

All vector preparations were examined at high (×1000) magnification using the same equipment, as for examination of blood smears. Parasite images were collected to prepare measurement. All measurements are in micrometres. Statistical analyses were carried out using ‘R studio’ v.3.4.3. Representative preparations of vector stages were deposited in the Nature Research Centre, Vilnius, Lithuania, under the accession numbers 49126–49133 NS.

### DNA extraction, PCR amplification, sequencing and sequence data analysis

Standard ammonium acetate protocol was applied for DNA extraction from blood and exposed biting midge samples [51]. A nested PCR protocol targeting a fragment of cytochrome *b* gene (*cytb*) was used [1, 52]. The first reaction was conducted with primers HaemNFI/HaemNR3 capable of amplifying *Haemoproteus*, *Plasmodium* and *Leucocytozoon* DNA. For the nested reaction, primers HaemF/HaemR2 capable of *Haemoproteus* and *Plasmodium* DNA amplification were used. Target DNA was amplified in 25 μl total volume including 50 ng of total genomic DNA template (2 μl), 12.5 μl of Dream Taq Master Mix (Thermo Fisher Scientific, Vilnius, Lithuania), 8.5 μl of nuclease-free water and 1 μl of each primer (10 μM concentration). One negative (nuclease-free water) and one positive control (a sample with *Haemoproteus* sp. infection positive in the slide) were used in every run. PCR products were evaluated using electrophoresis in a 2% agarose gel. Positive PCR products of ~480 bp were precipitated and sequenced from both strands using the Big Dye Terminator V3.1 Cycle Sequencing Kit (Thermo Fisher Scientific, Vilnius, Lithuania) and ABI PRISM™ 3100 capillary sequencing robot (Applied Biosystems, Foster City, CA, USA). Sequences were edited and aligned using BioEdit software [53] to create a consensus sequence. The presence of double peaks in electropherograms would have been considered as an indication of a mixed infection [54]. Consensus sequences obtained were aligned using BLAST (Basic Local Alignment Search Tool) with sequences from MalAvi (http://mbio-serv2.mbioekol.lu.se/Malavi/blast.html) and GenBank database (http//www.ncbi.nlm.nih.gov/BLAST). Sequences were deposited in GenBank (MN025422–MN025425).

Additionally, a longer *cytb* fragment (1001 bp) of each sample was amplified using a nested PCR protocol with primers AE298/AE299 for the first reaction, and primers AE064/AE065 for the second PCR reaction [55]. A fragment of caseinolytic protease (*clpc*) from the plastid genome (554 bp) was amplified using a nested protocol [56]. Sequences were deposited in the GenBank database under the accession numbers MK843310–MK843317. These data were not used in this study but are important for future taxonomic and phylogenetic work.

The possible presence of co-infections in parasite-donor birds was carefully checked, and the presence of co-infections was ruled out due to the absence of double peaks in sequence chromatograms and microscopic examination of blood films (see description of methods above). Importantly, presence of a single infection of corresponding parasite lineages was also supported by sequencing DNA isolated from insects when the sporogony was completed; double peaks in sequence chromatograms were absent.

### Phylogenetic analysis

Phylogenetic relationships among *Haemoproteus* species in which vectors have been identified was inferred using the Bayesian algorithm implemented by MrBayes v.3.2.0 [57]. In total, 52 haemosporidian *cytb* sequences were used, 40 belonging to *Haemoproteus* species, 11 to *Plasmodium* spp. and one to *Leucocytozoon* sp. (lSISKIN2) was used as outgroup. Generalised time-reversible (GTR) evolutionary model was selected by MrModeltest2 [58]. Two simultaneous runs were conducted with a sample frequency of every 100th generation over 3 million generations. We discarded 25% of the trees as ‛burn-inʼ. The remaining trees were used to construct a majority rule consensus tree. The phylogeny was visualized using Fig Tree v.1.4 [59].

### Preparations of *in vitro* zygotes and ookinetes of *Haemoproteus* species

**Fig. 1:**
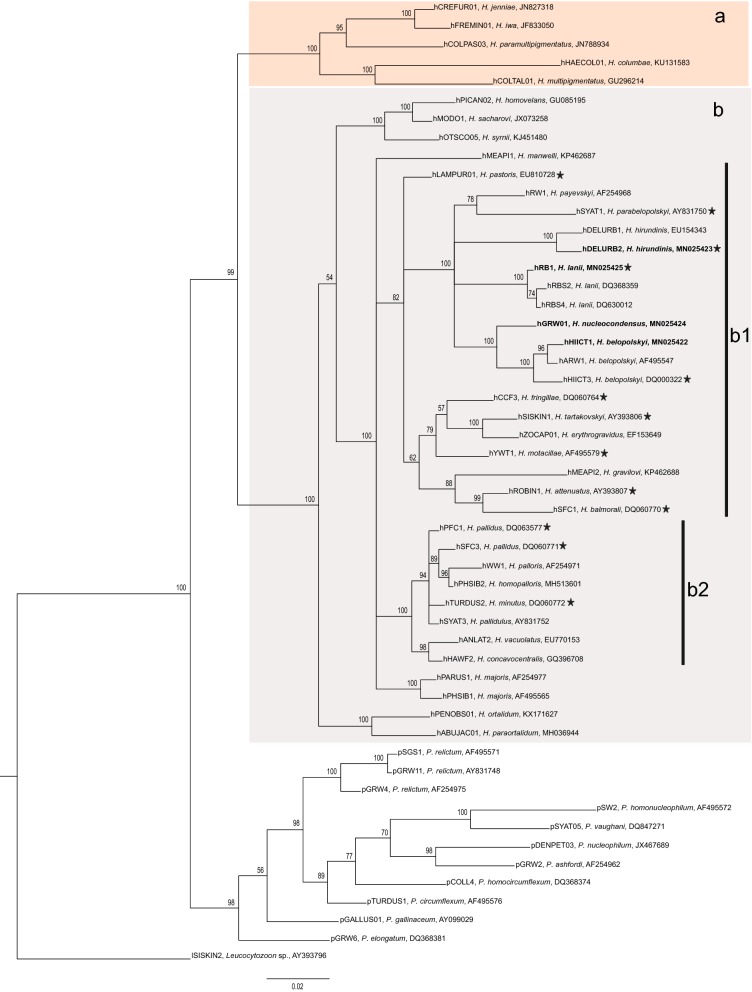
Bayesian phylogenetic inference of *cytb* lineages (478 bp) of 33 *Haemoproteus* species. The tree is rooted with *Leucocytozoon* sp. sequence (lSISKIN2). Clades A and B contain species of subgenera *Haemoproteus* and *Parahaemoproteus*, respectively. Sub-clade b1 contains species with slow development and relatively large-sized ookinetes, which produces a long outgrowth during development and possess prominent vacuoles in the cytoplasm. Sub-clade b2 contains species with fast development and relatively small ookinetes, which do not produce outgrowths and usually do not possess prominent vacuoles in the cytoplasm. MalAvi lineage codes are provided, followed by parasite species names and GenBank accession numbers. Nodal support values indicate Bayesian posterior probabilities. Names of the parasites, for which sporogony was investigated in this study are given in bold. Stars indicate parasite species, for which development *in vitro* has been investigated. Other explanations are given in the text

From the 27 *Haemoproteus* lineages shown in sub-clades b1 and b2 (Fig. 1), *in vitro* zygote and ookinete preparations were available for 13 of them (Fig. 1, indicated with a star). This material was collected and deposited in the collection of the Nature Research Centre, Vilnius (Lithuania) during *in vitro* studies by Valkiūnas [2] and Valkiūnas et al. [39, 40]. These preparations (Additional file 1: Table S1) were used in this study to compare the development of corresponding *Haemoproteus* species *in vitro*. These materials were examined as described above for blood smears.

## Results

The presence of single *Haemoproteus* infections was confirmed in all donor birds and these findings were supported both by microscopic examination of blood films and the absence of double-peaks in PCR-based testing. Exposed biting midges were also tested by the same PCR protocol used to test blood samples. It was possible to confirm the presence of the same parasite lineage in exposed insects and in blood samples, along with the absence of double-peaks in sequencing.

### *In vitro* exflagellation and ookinete development of *Haemoproteus hirundinis*

**Fig. 2:**
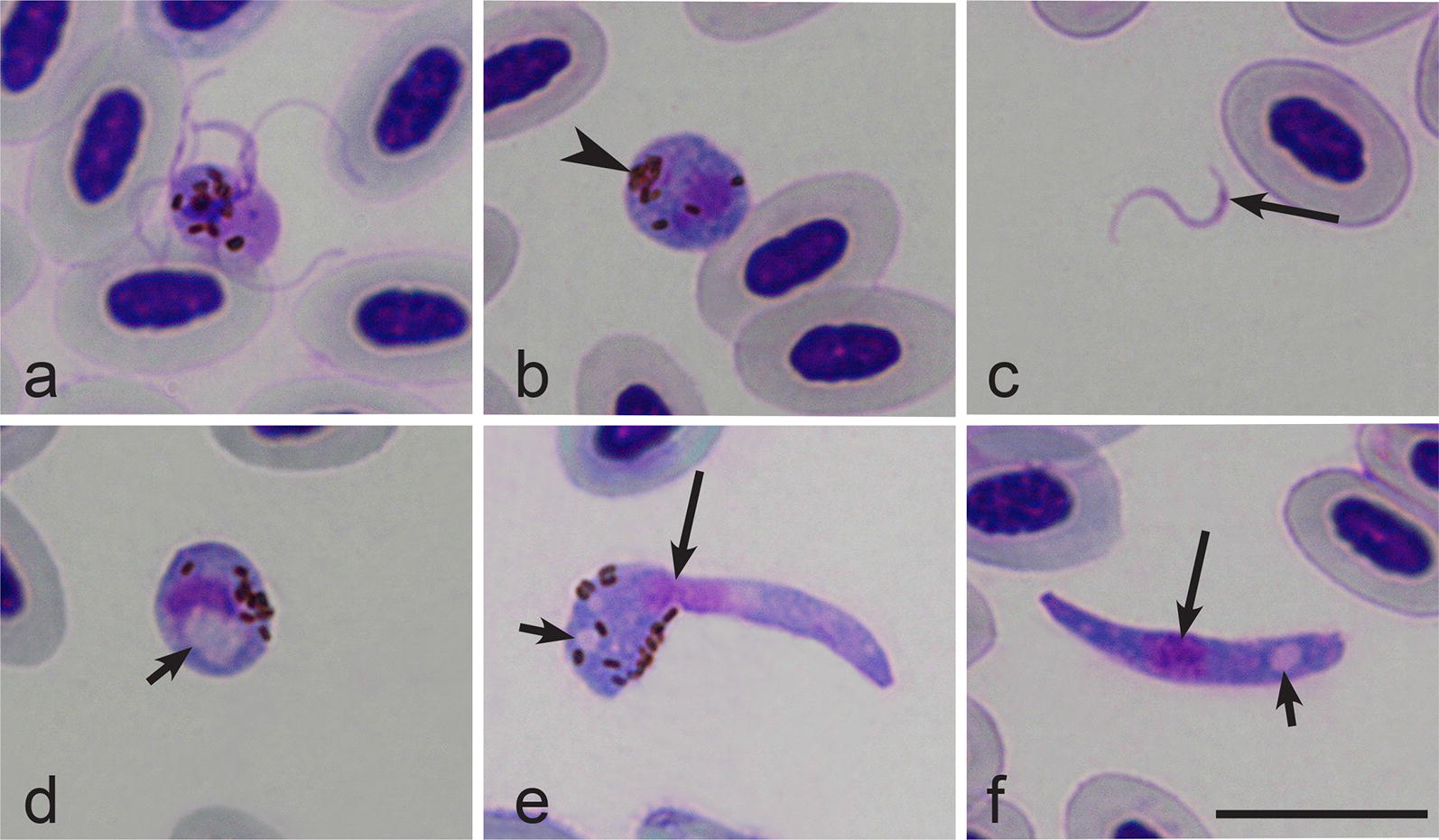
*In vitro* exflagellation (**a**), gametes (**b**, **c**), zygote (**d**) and ookinetes (**e**, **f**) of *Haemoproteus hirundinis* (hDELURB2). **a** Exflagellating microgametocyte. **b** Macrogamete. **c** Microgamete. **d** Zygote (note presence of a large vacuole in the cytoplasm). **e** Developing ookinete 3 h after exposure of mature gametocytes to air (note the presence of a long outgrowth and few vacuoles). **f** Mature ookinete with anterior end thinner than the posterior end (note the presence of a prominent vacuole, and absence of pigment granules). Long simple arrows, nuclei of parasites; short simple arrows, vacuoles; simple arrowhead, pigment granules. Methanol-fixed and Giemsa-stained thin films. *Scale-bar*: 10 µm

Within 5 min after exposure of blood to air (EBA), mature macrogametocytes rounded up and exflagellation of microgametes was observed (Fig. 2a). First macrogametes (Fig. 2b) were observed between 10–15 min EBA, and at this time free microgametes were also observed (Fig. 2c). Zygotes were observed 60 min after EBA; it was possible to distinguish them due to the presence of a prominent vacuole located close to the nucleus (Fig. 2d). The initial stages of ookinete differentiation were detected 3 h after EBA, when a long finger-like outgrowth appeared; the latter locates tangentially to the main body of the differentiating ookinete (Fig. 2e). As the ookinete develops, this outgrowth extends longitudinally and forms the anterior or apical end of the ookinete. The first fully grown ookinetes were observed between 6–8 h after EBA (Fig. 2f). At this stage of development, parasite usually does not possess pigment granules anymore, and usually two prominent vacuoles (one at the anterior and another at apical pole) were visible in the cytoplasm (Fig. 2f). Mature ookinetes of *H. hirundinis* (*n* = 21) measured 12.8–18.9 µm (on average 15.3 ± 1.6 µm) in length, 1.9–3.2 (2.5 ± 0.3) µm in width and 21.0–43.4 (28.9 ± 5.2) µm^2^ in area.

### Sporogonic development *in vivo*

**Fig. 3:**
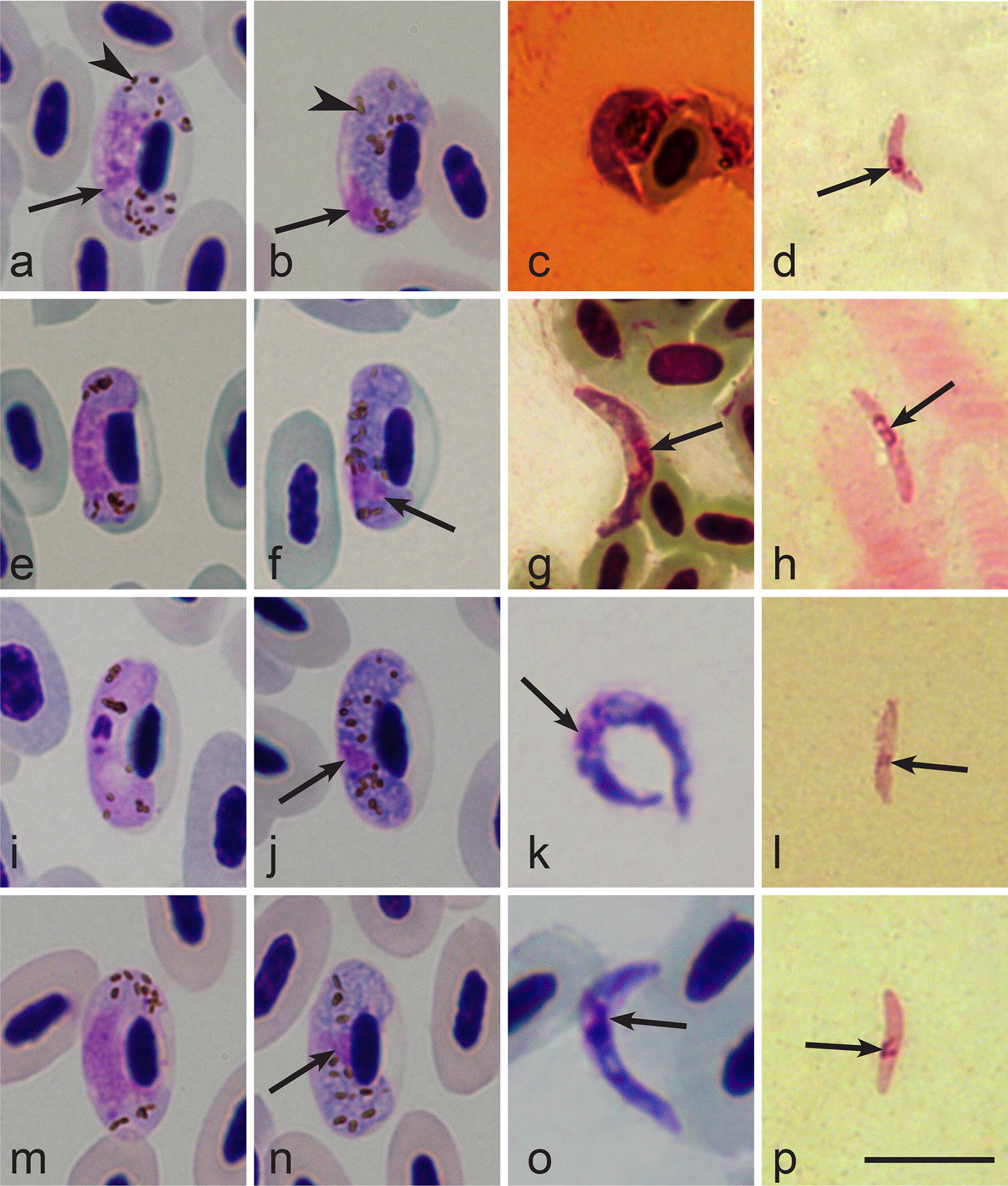
Mature gametocytes (**a**, **b**, **e**, **f**, **i**, **j**, **m**, **n**) from peripheral circulation of birds and ookinetes (**c**, **g**, **k**, **o**) and sporozoites (**d**, **h**, **l**, **g**) in *Culicoides nubeculosus*. **a**–**d** *Haemoproteus belopolskyi* (*cytb* lineage hHIICT1). **E**–**h** *Haemoproteus hirundinis* (hDELURB2). **i**–**l** *Haemoproteus nucleocondensus* (hGRW1). **m**–**p** *Haemoproteus lanii* (hRB1). Microgametocytes (**a**, **e**, **i**, **m**) and macrogametocytes (**b**, **f**, **j**, **n**) in the blood of *Hippolais icterina* (**a**, **b**), *Delichon urbicum* (**e**, **f**), *Acrocephalus arundinaceus* (**i**, **j**) and *Lanius collurio* (**m**, **n**). Long simple arrows, nuclei of parasites; simple arrowheads, pigment granules. Methanol-fixed and Giemsa-stained preparations. *Scale-bar*: 10 µm

The capacity of *C. nubeculosus* to support the sporogonic development of *H. belopolskyi* (hHIICT1), *H. hirundinis* (hDELURB2), *H. nucleocondensus* (hGRW1) and *H. lanii* (hRB1) was confirmed by microscopic examination of corresponding bird blood stages and insect preparations (Fig. 3). Sporozoites of all these species were detected in salivary gland preparations (Fig. 3d, h, l, p).

Mature ookinetes of all examined species were observed in midgut preparations between 6 and 8 h post-exposure (hpe). They were observed 8 hpe in *H. belopolskyi* preparations (Fig. 3c), 6–8 hpe in *H. hirundinis* (Fig. 3g), 6 hpe in *H. nucleocondensus* (Fig. 3k) and 7 hpe in *H. lanii* (Fig. 3o). A few morphologically non-deformed ookinetes of studied parasite species were observed. Their length and width measured 15.5 × 2.0 µm in *H. belopolskyi* (*n* = 1); 9.4 × 2.5 µm in *H. hirundinis* (*n* = 1); 12.9–18.6 × 1.3–2.1 (mean 15.8 ± 1.5 by 1.6 ± 0.3) µm in *H. nucleocondensus* (*n* = 17) and 9.4–14.4 × 1.2–2.2 (mean 12.6 ± 1.5 by 1.6 ± 0.3) µm in *H. lanii* (*n* = 8). Ookinetes of all species were worm-like elongate bodies, with approximately centrally located nuclei and vacuolated cytoplasm; pigment granules were not observed. *Haemoproteus nucleocondensus* ookinetes were often observed in a circular position, with both anterior and posterior ends almost touching each other, resembling a circle (Fig. 3k).

Sporozoites were observed in salivary glands preparations between 7–9 days post-exposure (dpe). They were detected 7–9 dpe in *H. belopolskyi* and *H. hirundinis* (Fig. 3d and h, respectively), 7–8 dpe in *H. nucleocondensus* (Fig. 3l) and 6–9 dpe in *H. lanii* (Fig. 3p). Measurements of *H. lanii* (*n* = 15) sporozoites were 6.0–9.0 (mean 7.3 ± 1.0) µm in length, 0.9–1.4 (1.1 ± 0.1) µm in width and 2.4–4.2 (3.6 ± 0.5) µm^2^ in area. A small number of sporozoites were observed in other species. Their length and width measured 6.7–8.0 × 1.1–1.3 µm in *H. belopolskyi* (*n* = 4), 10.0 × 1.1 µm in *H. hirundinis* (*n* = 1) and 8.3 × 1.4 µm in *H. nucleocondensus* (*n* = 1). Sporozoites of all four studied species were fusiform bodies with pointed ends and slightly off-centre located nuclei.

### Phylogenetic inference and its correspondence to patterns of ookinete development

All four *Haemoproteus* species investigated in this study clustered in a well-supported clade with other species transmitted by *Culicoides* insects (Fig. 1, Clade B). This clade contains parasites belonging to *Parahaemoproteus* subgenus. *Haemoproteus* (*Haemoproteus*) species, which are transmitted by louse flies (Hippoboscidae), clustered in a well-supported sister clade (Fig. 1, Clade A).

**Fig. 4:**
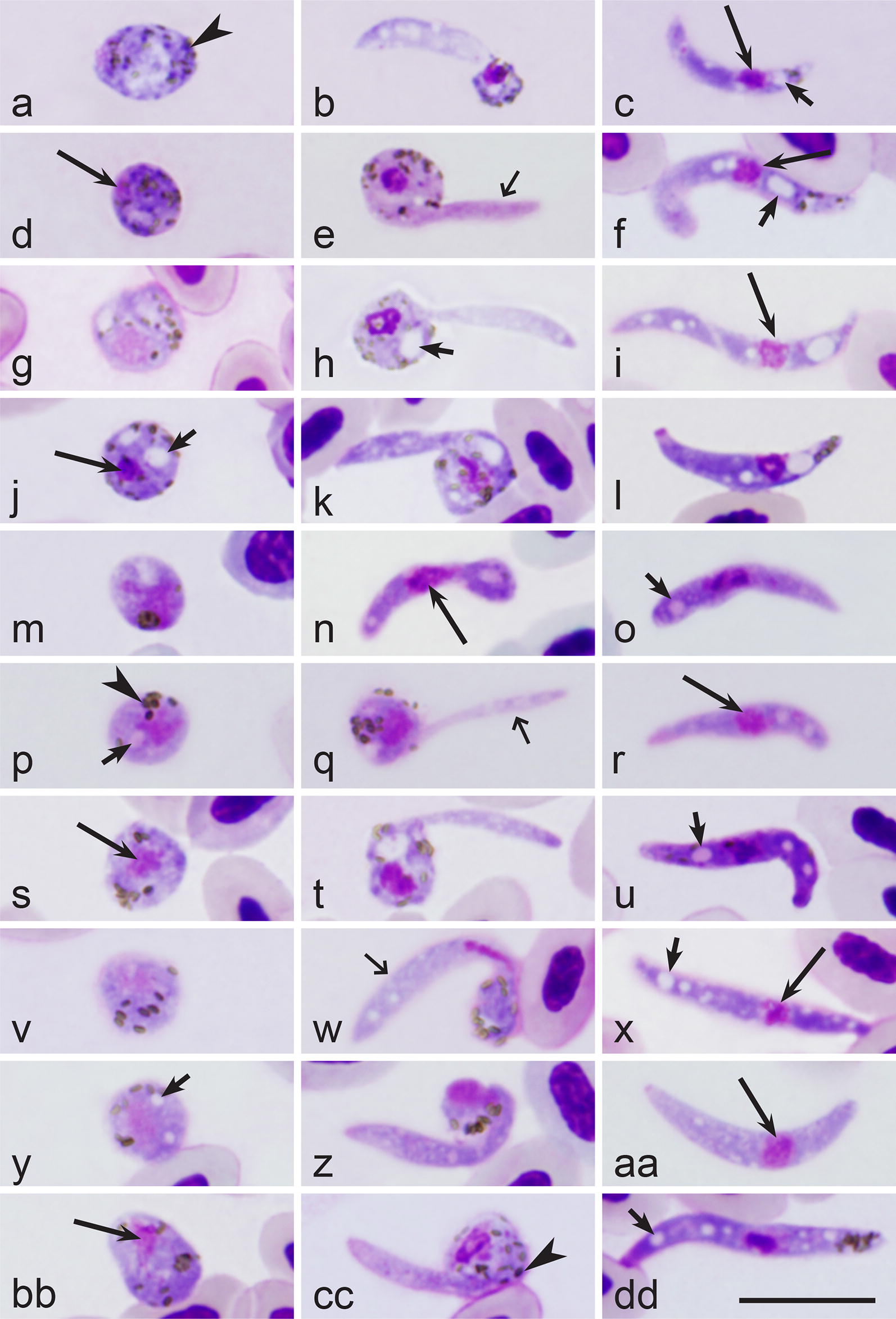
Zygotes (**a**, **d**, **g**, **j**, **m**, **p**, **s**, **v**, **y**, **bb**), growing ookinetes (**b**, **e**, **h**, **k**, **n**, **q**, **t**, **w**, **z**, **cc**) and mature ookinetes (**c**, **f**, **i**, **l**, **o**, **r**, **u**, **x**, **aa**, **dd**) of *Haemoproteus attenuatus* (*cytb* lineage hROBIN1, **a**–**c**), *Haemoproteus balmorali* (hSFC1, **d**–**f**), *Haemoproteus belopolskyi* (hHIICT3, **g**–**i**), *Haemoproteus fringillae* (hCCF3, **j**–**l**), *Haemoproteus hirundinis* (hDELURB2, **m**–**o**), *Haemoproteus lanii* (hRB1, **p**–**r**), *Haemoproteus motacillae* (hYWT1, **s**–**u**), *Haemoproteus parabelopolskyi* (hSYAT1, **v**–**x**), *Haemoproteus pastoris* (hLAMPUR1, **y**–**aa**) and *Haemoproteus tartakovskyi* (hSISKIN1, **bb**–**dd**) during development *in vitro*. Note that all these parasites produce long outgrowths during initial stages off ookinete development, and their ookinetes are markedly vacuolated; these parasites appeared in one relatively well-supported sub-clade b1 in the phylogenetic tree (Fig. 1). Long simple arrows, nuclei of parasites; short simple arrows, vacuoles; simple arrowheads, pigment granules; short simple wide arrows, outgrowths of developing ookinetes. Methanol-fixed and Giemsa-stained thin blood films. *Scale-bar*: 10 µm

**Fig. 5:**
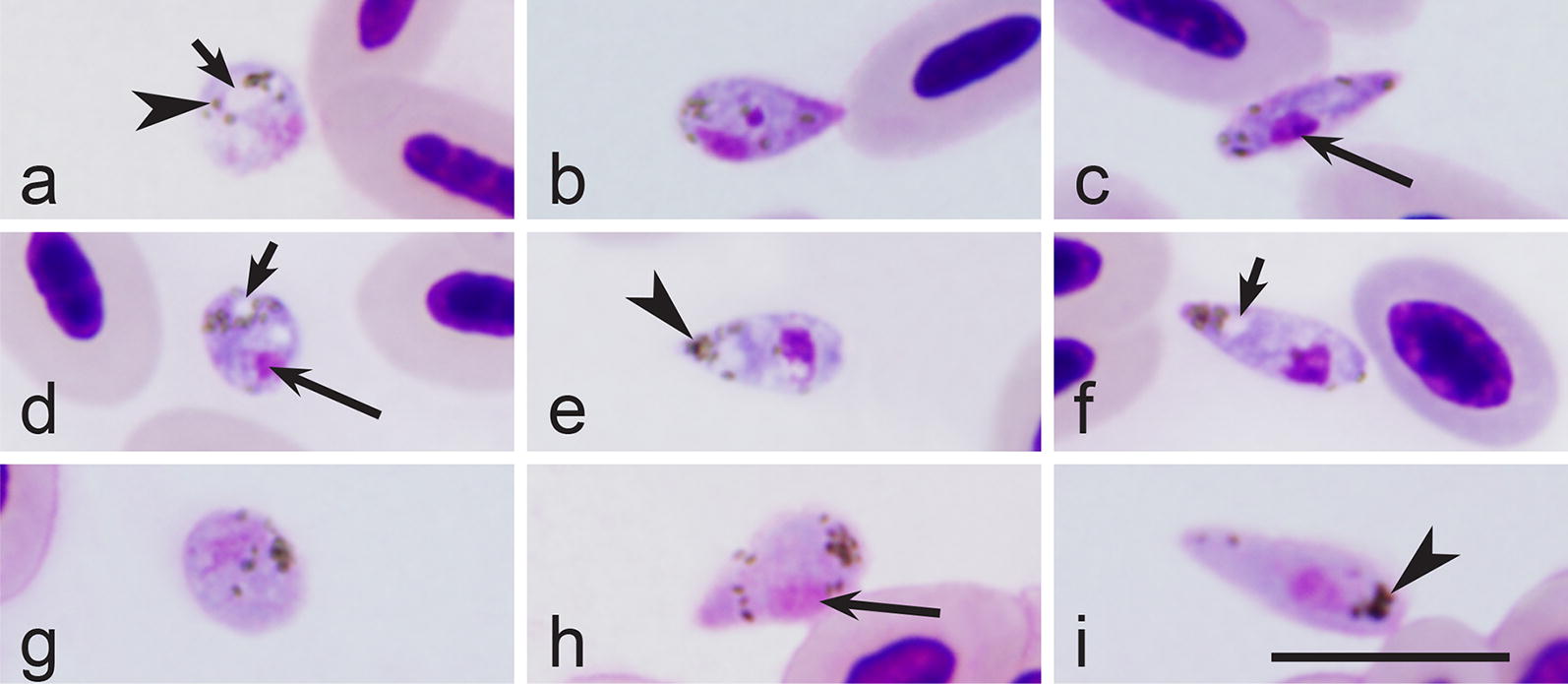
Zygotes (**a**, **d**, **g**), growing ookinetes (**b**, **e**, **h**) and mature ookinetes (**c**, **f**, **i**) of *Haemoproteus minutus* (hTURDUS2, **a**–**c**), *Haemoproteus pallidus* (hSFC3, **d**–**f**) and *Haemoproteus pallidus* (hPFC1, **g**–**i**) during development *in vitro*. Note that all these parasites do not produce any outgrowths during ookinete development, and their ookinetes do not contain vacuoles (**c**, **d**) or are only slightly vacuolated (**f**); these parasites appeared in one well-supported sub-clade b2 in the phylogenetic tree (Fig. 1). Long simple arrows, nuclei of parasites; short simple arrows, vacuoles; simple arrowheads, pigment granules. Methanol-fixed and Giemsa-stained thin blood films. *Scale-bar*: 10 µm

Fourteen identified species of haemoproteids clustered together in our phylogenetic analysis (Fig. 1, sub-clade b1). Sporogonic development has been investigated in ten of these parasites, and data about their ookinete development are available; all these parasites are characterised by the presence of long outgrows during initial stages of ookinete development, the readily distinguishable character (Fig. 4b, e, h, k, n, q, t, w, z, cc). Additionally, mature ookinetes of these parasites develop relatively slowly *in vitro*; they appear approximately 6 h after EBA and even later at ~20 °C [2]. Interestingly, two *Haemoproteus* parasites (*H. minutus* and *H. pallidus*), which do not produce long outgrowth on initial stages of ookinete development (Fig. 5b, e, h), clustered together and appeared in a different well-supported sub-clade (Fig. 1, sub-clade b2) separately from parasites of sub-clade b1 (Fig. 1). Ookinetes of these two parasites develop relatively fast *in vitro*; they appear approximately between 2 and 3 h after EBA at same conditions [2]. Because (i) the sub-clades b1 and b2 are relatively well supported and (ii) the parasites appeared in these clades are readily distinguishable both by the rate of development and the mode of ookinete transformation at the initial stage of development, both these characters should be of important phylogenetic value. In other words, phylogenies based on partial *cytb* reflect patterns of ookinete development in haemosporidian parasites.

## Discussion

This study adds four species of *Haemoproteus* to the list of avian haemoproteids that are transmitted by biting midges of the Ceratopogonidae and complete sporogonic development in laboratory-reared *C. nubeculosus*. The presence of sporozoites of *H. belopolskyi* (hHIICT1), *H. hirundinis* (hDELURB2), *H. nucleocondensus* (hGRW1) and *H. lanii* (hRB1) in the salivary glands of *C. nubeculosus* biting midges is evidence that this insect likely can act as a natural vector of these parasites.

Eight species of haemoproteids were recorded completing sporogonic development and producing sporozoites in *C. nubeculosus* [22, 23]. This insect readily supports sporogony of haemoproteids and is easy to maintain in the laboratory; we recommend using *C. nubeculosus* in experimental avian *Haemoproteus* research. This insect has a wide distribution across Eurasia [49] and can feed on the blood of many mammalian and avian species [20, 60–63].

Despite the simple morphology of sporozoites and few morphological characters visible under the light microscope in *Haemoproteus* species, it is possible to notice morphological differences among them on parasite species level [20–22]. Sporozoites of some species can be readily distinguished from each other by length, as is the case in *Haemoproteus noctuae* and *Haemoproteus syrnii* during development in *Culicoides impunctatus*; mainly, sporozoites of the former parasite are significantly longer [20]. In the present study, sporozoites of *H. hirundinis* seem to be longer than in other species as well, while the width was similar in all studied species. However, this information should be carefully interpreted because sporozoites of *Haemoproteus* parasites were few in preparations during this study. Only preparations of *H. lanii* contained sufficient numbers of sporozoites to perform statistical analysis, providing opportunity to measure and analyse their morphological features in more detail.

It is interesting to note that only a few sporogonic stages were found in the majority of insect preparations of almost all investigated parasite species in this study. This prevented a detailed statistical comparison of reported ookinetes, oocysts and sporozoites. The low intensity of sporogonic stages is likely due to the selection of donor birds with light parasitaemia of mature gametocytes (approximately 0.1%) for exposure of insects in this study. *Haemoproteus* parasites are highly virulent and even lethal to *C. nubeculosus* and other biting midges and even mosquitoes when they feed on a blood meal with gametocytaemia over 2–3% [11, 12]. Numerous ookinetes of *Haemoproteus* parasites develop rapidly, then migrate to midguts and kill insects due to midgut damage after feeding on heavily infected blood. It is thus essential to avoid such conditions during experiments aimed at studying complete sporogonic development of *Haemoproteus* parasites in vectors. Several studies used donor birds with gametocytaemia ranging between 0.2% and approximately 2% [10, 63] to expose biting midges. Mortality of exposed insects occurred, but some insects survived and more ookinetes, oocysts and sporozoites were observed in insect preparations for analysis. Thus, it is important to determine optimal parasitaemia, which can be used in experimental exposure of insects: numerous insects would survive, but parasite sporogonic stages might be difficult to find and visualize if the parasitaemia is too light (0.1% in this study). Conversely, the majority of insects would die soon after exposure if the parasitaemia is relatively high (> 1%), but more sporogonic stages would be detected in the few surviving insects. In each vector exposure experiment, the optimal parasitaemia in donor birds should be determined. Based on available information, it is likely between 0.5 and 1% in many tested *Haemoproteus* and *Culicoides* species. Actually, this is close to *Haemoproteus* parasitaemia, which predominates in wildlife and might be optimal for natural transmission [2]. Because the rate of ookinete development and ookinete size are markedly different in different *Haemoproteus* species [23], virulence of haemosporidian parasites to their insect vectors probably depends on certain parasite and insect species. However, this issue remains insufficiently understood. Further studies using birds with different levels of parasitaemia should be encouraged to determine the best parasitaemia range for experimental infections, so that insects would survive during the experiment and sufficient number of ookinetes, oocysts and sporozoites can be obtained for research in insect preparations depending on each study aim.

Only a small portion of *Plasmodium* spp. sporozoites reach salivary glands after maturation of oocysts. In *Plasmodium gallinaceum*, an avian malarial parasite, about 10–20% of sporozoites released from oocysts were located in *Aedes aegypti* salivary glands. This migration happened during eight hours after sporozoites were released into the haemocoel [64]. After this period, the sporozoites persisted in the haemocoel for a short period until degradation [64, 65]. It is possible that the same pattern of sporozoite survival and salivary gland invasion occurs in *Haemoproteus* parasites, but such observations remain insufficient in haemoproteids. It is known that almost all insects fed on birds infected with *Haemoproteus* species can support ookinetes development, but only about 20% of them support oocysts’ development and less than half of infected insects that survived until termination of sporogony possessed sporozoites in salivary gland preparations of *C. impunctatus* [10]. Because a significantly lower number of sporozoites (usually < 100) develop in oocysts of *Haemoproteus* (*Parahaemoproteus*) parasites than in avian *Plasmodium* species (usually > 1000) [2], few of the former probably reach salivary glands, resulting in difficulties to visualize them in preparations. This also can explain the low number of sporozoites observed in exposed *C. nubeculosus* in this study.

*In vitro* exflagellation and development of ookinetes have been examined in several *Haemoproteus* species [2, 37–39, 41]. The present study adds information about *in vitro* development of *H. hirundinis*, a common parasite of swallows. Regarding experimental vector studies, these data are important because they show the rate of mature ookinete development, indicating the most optimal time interval when insects should be dissected for ookinete detection *in vivo* during experimental research. After maturation, ookinetes of some *Haemoproteus* species rapidly escape from midgut contents and penetrate epithelial cells of the gut, so they can be overlooked in the midgut in *in vivo* preparations if ookinete preparations were made too late and the ookinetes had already moved from the midguts [31]. The rate of ookinete development is markedly different in different *Haemoproteus* species: it ranges from ~2 hpe in *H. minutus* to 6–12 hpe and even more in *H. tartakovskyi* (between 6 and 24 hpe) at ~20 °C [2, 63]. As a result, ookinetes of *H. minutus* are absent from midgut preparations prepared 4 hpe and later after exposure and cannot be detected, but ookinetes of *H. tartakovskyi* are still developing and would be readily visible. Our study shows that ookinete preparations of *H. hirundinis* should be prepared approximately 6 hpe at 20 °C. For many examined species, the most optimal periods for visualization of *Haemoproteus* parasites in vectors are 6–8 hpe for ookinetes, 3–5 dpe for oocysts and 6–8 dpe for sporozoites [12, 22, 23, 63].

Sporogonic development of *H. belopolskyi* (hHIICT1) has been investigated in wild-caught biting midges *C. impuctatus* at 15–18 °C; ookinetes were detected 1 dpe and sporozoites 7 dpe [31]. In the present study, the sporogonic development of the same parasite lineage was more rapid: mature ookinetes were recorded 8 hpe and sporozoites were observed 6 dpe. This difference might be due to the different temperature conditions for sporogony; the insects were maintained at 24–25 °C during this study. Numerous studies addressed the relationship of sporogony in *Plasmodium* parasites and temperature conditions [66], but information about *Haemoproteus* species remain insufficient.

In a previous study with *H. lanii*, ookinetes and sporozoites were observed in experimentally exposed *C. impunctatus* 1–2 and 5–8 dpe, respectively; the exposed insects were kept at natural wildlife temperature conditions ranging between 14 °C at night and 18 °C during the daytime [10]. In the present study, ookinetes of *H. lanii* hRB1 were found 6 hpe and sporozoites detected 6–9 dpe at 24–25 °C. In both studies the development rate of sporozoites was similar, despite the difference in temperature conditions. The reported difference between the sporogony rate in these two studies of *H. lanii* is likely due to different intervals of exposed insect dissections: sporozoites were detected 5 dpe in the former study, but dissections of insects for sporozoites were performed one day later in this study. However, it is important to note that three lineages of *H. lanii* have been identified, all of which are present in the same species of avian host (*Lanius collurio*) [67]. The study by Valkiūnas & Iezhova [10] was carried out using an unidentified lineage of *H. lanii*, so it is difficult to rule out that observed differences are due to different parasite lineages used in the experiments. Futher detailed experimental research is needed to understand *Haemoproteu*s parasite sporogony rate in different parasite lineages belonging to the same species.

As expected, all four parasite lineages used in this study clustered together (Fig. 1, sub-clade b1) in our phylogenetic analysis and appeared with other *Haemoproteus* (*Parahaemoproteus*) species that are transmitted by biting midges (Fig. 1, Clade B) [20]. All species belonging to the subgenus *Haemoproteus* and transmitted by louse flies of the Hippoboscidae [2] were placed in a well-supported sister clade (Fig. 1, Clade A). This phylogenetic analysis is in accordance with studies, which showed that phylogenies using *cytb* indicate parasite-vector relationships. This gene is important for the metabolism of haemosporidians in vectors and is of phylogenetic value in evolutionary studies aiming determination of major Dipteran insect groups, which are involved in haemosporidian parasite transmission [22, 68]. Phylogenies based on this gene readily distinguish parasites belonging to subgenera *Haemoproteus* and *Parahaemoproteus* [20–22, 56, 69]. It is interesting to note that the differences between species of *Haemoproteus* and *Parahaemoproteus* are not only due to inhabiting different groups of dipteran insects (louse flies and biting midges, respectively), but also are manifested in the size of oocysts, the number of developing germinate centres in oocysts and the number of sporozoites produced in each of these groups [2]. In addition, our study shows that phylogenies based on partial *cytb* also indicate patterns of ookinete development in haemosporidian parasites (Fig. 1).

Maturing *H. hirundinis* ookinetes possess long finger-like outgrowths (Fig. 2e). This readily distinguishable character has been observed in developing ookinetes of several haemoproteid parasites [2, 37, 40] such as *Haemoproteus attenuatus* (hROBIN1) (Fig. 4a–c), *Haemoproteus balmorali* (hSFC1) (Fig. 4d–f), *H. belopolskyi* hHIICT3 (Fig. 4g–i), *Haemoproteus fringillae* hCCF3 (Fig. 4j–l), *H. hirundinis* (hDELURB2) (Fig. 4m–o), *H. lanii* (hRB1) (Fig. 4p–r), *Haemoproteus motacillae* (hYWT1) (Fig. 4s–u), *Haemoproteus parabelopolskyi* (hSYAT1) (Fig. 4v–x), *Haemoproteus pastoris* (hLAMPUR1) (Fig. 4y–aa) and *Haemoproteus tartakovskyi* (hSISKIN1) (Fig. 4bb–dd). Marked vacuolization of the cytoplasm also seems to be a character of all species listed above (Fig. 4c, f, i, l, o, r, u, x, aa, dd); however, this feature does not seem to be a unique for parasites of this clade (Fig. 1, sub-clade b1), since vacuoles were also present in ookinetes of *H. pallidus* (lineage hSFC3) (Fig. 1, sub-clade b2 and Fig. 5e–f).

This study not only strengthens the conclusion of former studies that phylogenies based on partial *cytb* sequences readily distinguish lineages belonging to parasites of the subgenera *Haemoproteus* and *Parahaemoproteus* [20–22, 56, 69], but also shows that well-supported clades in such phylogenetic trees have important biological meaning. In particular, our study suggests that patterns of ookinete transformation from zygote stage can be forecasted based exclusively on such phylogenies, which are easy to develop. Additionally, the relatively small density of cellular organelles in macrogametocytes is indicated by pale staining of the cytoplasm in these sexually-important cells, and the pale staining is readily visible under a light microscope. This feature is likely of important phylogenetic value because all investigated haemoproteids possessing the same character cluster together in available phylogenetic trees, as has been reported in several previous studies [23, 70, 71]. Interestingly, ookinetes of parasites of this clade (*H. minutus*, *H. pallidus*) develop fast (≤ 4 hpe) at a temperature of approximately 18–20 °C. Our study extends this conclusion and shows that 14 parasite species, which appeared in a relatively well-supported sub-clade b1 (Fig. 1) also are similar due to patterns of their ookinete development. In general, in all these species (i) long finger-like outgrowths appear at early stage of ookinete development (Figs. 2e; 4b, e, h, k, n, q, t, w, z, cc); and (ii) ookinetes mature relatively slowly (≥ 6 hpe) at a temperature of approximately 18–20 °C. In summary, our study shows that phylogenies based on *cytb* and mitochondrial genomes not only indicate large groups of vectors, which transmit parasites of certain groups of haemosporidians [22, 69], but also group haemosporidian species based on delicate patterns of their sporogony [23].

The simple characters of haemosporidian ookinete development, which are readily recognisable under a light microscope (rate and mode of transformation) should be genetically determined, particularly due to the following reasons. First, complex morphological and functional changes precede each ookinete development, including building of organelles of the apical complex, endoplasmic reticulum, crystalloid particles and others [2, 72]. Transformation of ookinetes *via* development of long finger-like outgrowths is related to ultrastructural changes in zygotes; this process is genetically determined [73]. Secondly, the rate of ookinete development depends on many internal and external (temperature, insect gut-factors) structural circumstances mentioned above, and is also likely determined genetically. The present study shows that phylogenies based on mitochondrial genomes are worth more attention in regard to better understanding patterns of sporogonic development in haemosporidian parasites. Additionally, it is possible that *cytb* phylogenetic inferences are more likely to reflect haemosporidian parasite development in vectors rather than in blood and tissue stages. This can be explained by the critical role of mitochondrial complex during the development of haemosporidians in insect vectors [68].

The presence of more or less prominent vacuoles in the cytoplasm of ookinetes (Figs. 4 and 5) is a common feature of parasites in sub-clade b1 (Fig. 1). Such vacuoles also were observed in ookinetes of parasites belonging to subgenus *Haemoproteus* (Fig. 1, clade A) [38]. These cytoplasmic vacuole-like inclusions are in fact the gatherings of an amorphous dense material called crystalloid material [2, 74]. The crystalloid material is likely washed out during fixation with alcohols and looks like an empty space, which is usually described as a ‘vacuole’ in stained preparations under a light microscope. The crystalloid likely performs energy functions and takes part in the metabolism of lipids, which is genetically determined [2, 73–75]. However, there is a variation in the presence of these vacuoles even among different lineages of the same parasite species. This can be observed in *H. pallidus* (hSFC3) that possess vacuoles (Fig. 5d–f) and *H. pallidus* (hPFC1) that does not possess visible vacuoles in the ookinetes (Fig. 5g–i). The lack of vacuoles was registered in *H. minutus* (Fig. 5a–c). Available data indicated that ookinetes of parasites of sub-clade b1 (Figs. 1 and 4) are more heavily vacuolated than ookinetes of sub-clade b2 (Figs. 1 and 5). However, only two *Haemoproteus* species (*H. pallidus* and *H. minutus*) have been investigated in regard to this character so far, and additional studies are needed to prove or reject this hypothesis.

The transmission of *H. hirundinis* and *H. nucleocondensus* have not been reported in Europe [2, 48, 76]. These are common parasites of the northern house martin *D. urbicum* and the great reed-warbler *A. arundinaceus*, respectively. These birds become infected at African wintering grounds, but juveniles are free of these infections in Europe before seasonal migration. It has been speculated that absence of susceptible vectors might interrupt transmission. The present study rejects this hypothesis because sporogony of both parasites was completed in *C. nubeculosus*, which is widespread in Europe and readily bites birds ([49]; this study). It is interesting that the same epidemiology has been reported for *Plasmodium relictum* (pGRW4) infection in temperate regions of Europe. In general, vector and sufficient temperature conditions for sporogony of this parasite are present, but transmission is absent in Europe [41]. It is probable that other ecological factors, which are related to vector ecology, might restrict transmission of these haemosporidian infections at bird breeding grounds. The first factor might be associated with the nesting biology of these bird species. The northern house martin builds closed nests with a small entrance; such nests might be non-attractive for biting midges. However, information about this aspect of the biology of *Culicoides* biting midges remains insufficient. It is known that biting midges can visit artificially-made nest-boxes of forest birds [77], but it is unclear if the same happens in northern house martin nests. These birds often build the nests below human-made structures such as bridges and houses in densely populated human settlements at different ecological conditions. This is not the case for the great reed-warbler: this bird builds open nests. However, both the northern house martin and the great reed-warbler prefer to nest in relatively open areas either close to human settlements or in reeds, respectively [78, 79]. The latter bird species also nests in environments with windy conditions, which do not favour the long flight of fragile biting midges [80]. These ecological factors might minimize the contact of *Culicoides* biting midges with these two-bird species resulting in a low probability for infection transmission from parent birds to offspring. Additionally, the prepatent development of *H. hirundinis* and *H. nucleocondensus* remain non-investigated; there is no information about longevity of prepatent period during these infections. In some *Haemoproteus* species the prepatent period varies between two and three weeks [2]. The majority of northern house martin and great reed warbler populations leave for wintering grounds early (in August) in Europe when the majority of *H. hirundinis* and *H. nucleocondensus* infections might be still non-patent, and that might prevent determining infected juveniles before seasonal migrations, at least in some of them. The present study indicates that factors other than availability of vectors might restrict transmission of *H. hirundinis* and *H. nucleocondensus* in Europe. Ecological factors preventing transmission of avian haemosporidians need more research. Further detailed studies on parasite life-cycles and their vector ecology are needed for a better understanding the transmission of these infections and the epidemiology of haemoproteosis.

Partial sequences of *cytb* gene have often been used to barcode haemosporidians [67] and are also often used to develop phylogenies, which distinguish haemosporidians belonging to different families and genera [3, 15, 17, 37, 70. 71]. However, information about other gene sequences of avian haemosporidians remains insufficiently developed and difficult to use in phylogenetic analysis but is important in biodiversity research. This is particularly true for apicoplast gene sequences, which provide valuable information in distinguishing morphologically similar haemosporidian species, which differ just in a few nucleotides in partial *cytb* sequences [81, 82]. We added information about partial sequences of the *clpc* genes along with *cytb* gene sequences, which extend molecular characterization of studies haemosporidian species and might be helpful for future research.

## Conclusions

This study adds four species of avian haemoproteids (*H. belopolskyi* hHIICT1, *H. hirundinis* hDELURB2, *H. nucleocondensus* hGRW01 and *H. lanii* hRB1) to the list of parasites, which complete sporogony in *C. nubeculosus*. Due to the high virulence of avian haemoproteids in blood-sucking arthropods, parasite donor birds with light parasitaemia (of approximately 0.5–1%) are recommended to be used during experimental exposure of insects. Phylogenies based on partial sequences of *cytb* indicate patterns of sporogony of certain lineages. This study identified two clades of *Haemoproteus* lineages, which differ markedly in the rate of ookinete development and morphological patterns of ookinete transformation at initial stages of development. Ecological factors other than vector availability and temperature conditions should be identified to understand mechanisms preventing distribution of *H. hirundinis* and *H. nucleocondensus* infections and other haemosporidian parasites of tropical origin in temperate Europe.

## Supplementary information

**Additional file 1: Table S1.** Parasites, host species and accession numbers of preparations, which were used for comparisons of *in vitro* ookinete development in this study.

## Data Availability

The data supporting the findings of this study are included within the article and its additional file. Representative preparations of blood (Accession Numbers 49134NS-49147NS) and vector stages (49126NS-49133NS) were deposited in the Nature Research Centre, Vilnius, Lithuania. The sequences were deposited in the GenBank database under the Accession Numbers MN025422–MN025425 and MK843310–MK843317.

## Abbreviations

*cytb*: mitochondrial cytochrome b
DNA: deoxynucleic acid
dpe: days post-exposure
EBA: exposure of blood to air
hpe: hours post-exposure
PCR: polymerase chain reaction
