# Supplementary material for: Sporogony of four Haemoproteus species (Haemosporida: Haemoproteidae), with report of in vitro ookinetes of Haemoproteus hirundinis: phylogenetic inference indicates patterns of haemosporidian parasite ookinete development
Source: Parasit Vectors. 2019 Aug 28;12:422. doi: 10.1186/s13071-019-3679-1 (PMC6714444; doi:10.1186/s13071-019-3679-1)
Supplement: Supplementary file 1 — Additional file 1: Table S1. Parasites, host species and accession numbers of preparations, which were used for comparisons of in vitro ookinete development in this study. [file 13071_2019_3679_MOESM1_ESM.docx]

**Additional file 1: Table S1. Parasites, host species and accession numbers of preparations, which were used for comparisons of *in vitro* ookinete development in this study.**

| Parasites species (lineage) | Host species (Common name) | Accession number^a^ |
| --- | --- | --- |
| *Haemoproteus attenuatus* (hROBIN1) | *Saxicola rubetra* (Whinchat) | 49080-49083 NS |
| *Haemoproteus balmorali* (hSFC1) | *Luscinia luscinia* (Thrush nightgale) | 49084-49087 NS |
| *Haemoproteus belopolskyi* (hHIICT3) | *Hippolais icterina* (Icterine warbler) | 49088-49091 NS |
| *Haemoproteus fringillae* (hCCF3) | *Fringilla coelebs* (Common chaffinch) | 49092-49095 NS |
| *Haemoproteus hirundinis* (hDELURB2) | *Delichon urbicum* (Northern house martin) | 49096-49099 NS |
| *Haemoproteus lanii* (hRB1) | *Lanius collurio* (Red-backed shrike) | 49100-49101 NS |
| *Haemoproteus motacillae* (hYWT1) | *Motacilla flava* (Western yellow wagtail) | 49104-49107 NS |
| *Haemoproteus parabelopolskyi* (hSYAT1) | *Sylvia atricapilla* (Eurasian blackcap) | 49110-49113 NS |
| *Haemoproteus pastoris* (hLAMPUR1) | *Sturnus vulgaris* (Common starling) | 49114-49117 NS |
| *Haemoproteus tartakovskyi* (hSISKIN1) | *Carduelis spinus* (Eurasian siskin) | 49118-49121 NS |
| *Haemoproteus minutus* (hTURDUS2) | *Turdus merula* (Eurasian blackbird) | 49102-49103 NS |
| *Haemoproteus pallidus* (hSFC3) | *Muscicapa striata* (Spotted flycatcher) | 49108-49109 NS |
| *Haemoproteus pallidus* (hPFC1) | *Ficedula hypoleuca* (Eurasian pied flycatcher) | 49122-49125 NS |

^a^ Accession numbers at Nature Research Centre, Vilnius, Lithuania
